# The Activities of Current Antimalarial Drugs on the Life Cycle Stages of *Plasmodium*: A Comparative Study with Human and Rodent Parasites

**DOI:** 10.1371/journal.pmed.1001169

**Published:** 2012-02-21

**Authors:** Michael Delves, David Plouffe, Christian Scheurer, Stephan Meister, Sergio Wittlin, Elizabeth A. Winzeler, Robert E. Sinden, Didier Leroy

**Affiliations:** 1 Department of Life Sciences, Imperial College London, London, United Kingdom; 2 Genomics Institute of the Novartis Research Foundation, San Diego, California, United States of America; 3 Swiss Tropical & Public Health Institute Swiss TPH, Basel, Basel, Switzerland; 4 University of Basel, Basel, Switzerland; 5 The Scripps Research Institute, La Jolla, California, United States of America; 6 Medicines for Malaria Venture, Geneva, Switzerland; Walter and Eliza Hall Institute of Medical Research, Australia

## Abstract

**Background:**

Malaria remains a disease of devastating global impact, killing more than 800,000 people every year—the vast majority being children under the age of 5. While effective therapies are available, if malaria is to be eradicated a broader range of small molecule therapeutics that are able to target the liver and the transmissible sexual stages are required. These new medicines are needed both to meet the challenge of malaria eradication and to circumvent resistance.

**Methods and Findings:**

Little is known about the wider stage-specific activities of current antimalarials that were primarily designed to alleviate symptoms of malaria in the blood stage. To overcome this critical gap, we developed assays to measure activity of antimalarials against all life stages of malaria parasites, using a diverse set of human and nonhuman parasite species, including male gamete production (exflagellation) in *Plasmodium falciparum*, ookinete development in *P. berghei*, oocyst development in *P. berghei* and *P. falciparum*, and the liver stage of *P. yoelii*. We then compared 50 current and experimental antimalarials in these assays. We show that endoperoxides such as OZ439, a stable synthetic molecule currently in clinical phase IIa trials, are strong inhibitors of gametocyte maturation/gamete formation and impact sporogony; lumefantrine impairs development in the vector; and NPC-1161B, a new 8-aminoquinoline, inhibits sporogony.

**Conclusions:**

These data enable objective comparisons of the strengths and weaknesses of each chemical class at targeting each stage of the lifecycle. Noting that the activities of many compounds lie within achievable blood concentrations, these results offer an invaluable guide to decisions regarding which drugs to combine in the next-generation of antimalarial drugs. This study might reveal the potential of life-cycle–wide analyses of drugs for other pathogens with complex life cycles.

*Please see later in the article for the Editors' Summary*

## Abstract 2

Michael Delves and colleagues compare the activity of 50 current and experimental antimalarials against liver, sexual blood, and mosquito stages of selected human and nonhuman parasite species, including *Plasmodium falciparum*, *Plasmodium berghei*, and *Plasmodium yoelii*.

## Editors' Summary

**Background:**

Malaria is a life-threatening disease caused by the *Plasmodium* parasite, which is transmitted to people through the bites of infected mosquitoes. According to latest global estimates, about 250 million people are infected with malaria every year with roughly 800,000 deaths—most occurring among young children living in Africa. Malaria also causes severe morbidity in children, such as anemia, low birth weight, and neurological problems, which compromise the health and development of millions of children living in malaria endemic areas. In addition to strategies that scale up and roll out the prevention of malaria, such as country-wide programs to provide insecticide-treating bednets, in the goal to eradicate malaria, the global health community has refocused efforts on the treatment of malaria, including finding new compounds that target different stages of the parasite life cycle as it passes from vector to host and back.

The interruption of malaria transmission worldwide is one of the greatest challenges for the global health community. In January 2011, this journal published a series on The Malaria Eradication Research Agenda (malERA), which described a set of research and development priorities, identified key knowledge gaps and the necessary tools needed, and introduced a draft research and development agenda for the worldwide eradication of malaria.

**Why Was This Study Done?:**

Most currently available antimalarial drugs primarily target the disease-causing parasites' stages in the human blood system. But to eradicate malaria, new drugs that block transmission of the parasite between the human host and the mosquito vector, and eliminate the various stages of the parasite during its cycle in the human body, are needed. In this laboratory study, the researchers compared the profiles of all available and experimental antimalarials and analyzed each drug for activity against each specific stage in the malaria parasite's life cycle to provide a reference set of methods and data, that might serve as a benchmark to help guide the malaria research community in assessing the potential of newly discovered antimalarials. Furthermore, this analysis could provide insights into which chemical drug classes might provide transmission-blocking capabilities—an essential component of malaria eradication.

**What Did the Researchers Do and Find?:**

The researchers used novel laboratory techniques under standardized conditions to develop a series of novel assays to analyze the activities of 50 antimalarial compounds (current drugs and those under development) against three *Plasmodium* species encompassing every major cellular strategy of the malarial life cycle including drug resistant parasite strains. In their comparative analysis, the researchers undertook a chemical profiling approach to identify the drugs that block transmission from the host to the mosquito vector and additionally suppress transmission from the mosquito to the human host.

The researchers highlighted some encouraging results; for example, the potencies of some antimalarials against the asexual blood stage of cultivated *P. falciparum* and *P. vivax* isolates show a very good correlation, suggesting that most of the pathways inhibited by antimalarials in *P. falciparum* may also be valid targets in *P. vivax*. The researchers also have shown that approved drugs, such as pyronaridine and atovaquone, can target liver and sexual stages in addition to asexual blood stages. Furthermore, the researchers found promising results for new compounds currently in clinical trials, such as the endoperoxide OZ439, a stable synthetic molecule currently being studied in a phase IIa clinical trial, which seemed to be a strong inhibitor of gametocyte maturation and gamete formation. The new 8-aminoquinoline, NPC-1161B, also inhibited sporogony.

**What Do These Findings Mean?:**

The results of this analysis provide a valuable guide to help researchers decide which drugs and compounds show most promise as potential future antimalarial drugs for blocking the transmission of malaria. This study could also help researchers make decisions about which molecules could be best combined to provide the next generation of drugs that will succeed artemisinin compound therapy and support the eradication of malaria. Furthermore, this comprehensive approach to drug discovery could be applied to test drugs against other pathogens with complex life cycles.

**Additional Information:**

Please access these Web sites via the online version of this summary at http://dx.doi.org/10.1371/journal.pmed.1001169.

- The malERA a research agenda for malaria eradication sponsored collection, published by PLoS in January 2011, comprises 12 Review articles that discuss agendas in malaria research and development

## Introduction

Malaria remains one of the most widespread infectious diseases of our time. The latest estimates reveal that ∼250 million people are infected with malaria across the globe, of whom ∼800,000 die every year [1], the vast majority being young children. In 2007, the malaria eradication agenda was adopted by many researchers in the antimalarial community and target product profiles for new antimalarial medicines were defined [2]. Most available antimalarials were designed to target the pathogenic blood stages in humans and to address the constant threat of drug resistance [3]. However, to meet the objective of malaria eradication, medicines that block parasite transmission [4] and eliminate the asymptomatic and sometimes cryptic hepatic forms also need to be developed. The bottleneck populations of liver and sexual stage parasites [5] represent potential pathogen vulnerabilities that could be targeted by small molecules; the first such bottleneck is at the liver stage. Within minutes of being released by the bite of an infected female *Anopheles* mosquito, *Plasmodium* sporozoites reach the mammalian liver, where they invade hepatocytes and either lie dormant or develop over several days, eventually forming the schizonts that are the prelude to a blood stage infection [6]. Molecules that efficiently target the parasite stages in the liver would offer protection from infection and could theoretically eliminate the cryptic hypnozoite (dormant parasite) infection reservoirs formed by *P. vivax* and *P. ovale*. Because only 100 or so sporozoites may be introduced by a bite, there are likely to be many orders of magnitude fewer parasites at this stage than in an active blood stage infection, reducing the possibility of resistance arising. A second bottleneck occurs during sexual development. At each round of schizogony ∼1% of merozoites differentiate into gametocytes [7], and it is these developmentally arrested cells that are transmitted to the mosquito. Mature gametocytes are sexually dimorphic, forming microgametocytes and macrogametocytes that escape the red blood cell (RBC) and produce male and female gametes in the blood meal of the mosquito by processes known as exflagellation [8] and activation, respectively. Following fertilization the zygote differentiates into a motile and invasive ookinete within which the briefly diploid genome undergoes meiosis. These processes occur within an environment almost totally derived from host blood, which can therefore provide a novel and ideal conduit for the delivery of drugs to inhibit parasite transmission to the mosquito. Having crossed the mosquito midgut wall [9], the very few surviving ookinetes differentiate into oocysts, which undergo endomitosis, eventually producing thousands of daughter sporozoites. The sporozoites migrate from the midgut of the mosquito to its salivary glands where the lifecycle begins again.

Given that it would be highly desirable for candidate drugs to have activity against hepatic and sexual forms of the malarial parasite, it is surprising that few clinical trials, to date, have examined whether gametocyte carriage can be reduced following drug treatment. The only drugs found to be effective at reducing gametocyte carriage include artemisinin [10], artemisinin combination therapies (ACTs) [11],[12], methylene blue [13], and primaquine [12],[14]. Additionally, few studies have investigated the impact of drugs on the transmission of parasites from human blood to the mosquito vector [15]–[18], nor have many been designed to evaluate antihepatic stage activity. In the context of malaria eradication these gaps in our understanding of the full potential of the drug armoury are problematic.

Here we report the development of a series of novel assays against liver, sexual blood, and mosquito stages of the malaria parasite, using both drug-susceptible and drug-resistant parasite strains. We applied these assays to the current portfolio of schizonticidal compounds, consisting of 50 anti-infectives currently in use or under development..

## Methods

### Ethics Statement

All work involving laboratory animals for the host-to-mosquito transmission studies was performed in accordance with the European Union (EU) regulations “EU Directive 86/609/EEC” and within the regulations of the United Kingdom Animals (Scientific Procedures) Act 1986, sanctioned by UK Home Office Licence PLL70/6347 awarded in January 2008. Protocol design and implementation was guided by the principle of the three Rs (reduction, refinement, and replacement) and are of mild-to-moderate severity. Protocols are regularly reviewed and revised following approval by the Imperial College Ethics Review Committee.

### Parasite Maintenance

*P. berghei* parasites constitutively expressing GFP (PbGFPcon) [19]–[21] were routinely maintained as described previously [22]. Only blood showing exflagellating parasites was used in the transmission assays.

*P. falciparum* NF54 strain parasites were maintained in culture as described previously [23]. Gametocyte cultures were produced as described [24].

### *P. falciparum* In Vitro Antimalarial Activity

In vitro antimalarial activity was measured using the [^3^H]-hypoxanthine incorporation assay [25] with various strains of *P. falciparum* obtained from MR4. Results were expressed as the concentration resulting in 50% inhibition (IC_50_).

### *P. falciparum* Exflagellation Assay

Compounds were added to mature gametocyte of drug-sensitive *P. falciparum* cultures that showed the ability to exflagellate. After 24 h, exflagellation was triggered by a temperature decrease to ∼21°C and observed 20 min later under the microscope. Highly motile exflagellation centres were recorded for ∼50–150 adjacent fields of view and reported per 10,000 RBCs.

### *P. berghei* Ookinete Development Assay – Slide Method

Compounds and *P. berghei* gametocyte-infected blood were dispensed in a 96-well plate containing ookinete medium [26]. After 24 h at 19°C, Giemsa-stained ookinetes were counted under the microscope (×40) as previously described [27],[28].

### Standard Membrane Feed Assay – *P. berghei*

Membrane feeds were performed as described previously [22]. Briefly, PbGFPcon-infected mouse blood was mixed with compounds, immediately placed into membrane feeders (39°C) and offered for 30 min to 80–100 overnight-starved *A. stephensi* (SDA 500 strain). After 7–9 d at 19°C/80%RH, mosquito midguts were dissected out. Midguts were fixed with paraformaldehyde and oocyst number was determined microscopically by semi-automated analysis as previously described [29].

### Standard Membrane Feed Assay – *P. falciparum*

Gametocyte cultures were produced according to the same protocol as the Pf exflagellation assay. Pooled gametocyte cultures [30] were evenly divided between compounds in fresh medium and incubated at 37°C for 24 h. The parasite pellets were then resuspended in fresh RBCs and human serum treated with the compounds to a 50% haematocrit and immediately fed to mosquitoes as described above and then maintained at 27°C/60%RH for 10–12 d before dissection and counting.

### Liver Stage Assay

7.5×10^3^ HepG2 cells in 50 µl of medium (1.5×10^5^ cells/ml) were seeded in 384 well plates (Aurora 384 IQ-EB Black/Clear Plates) 20–26 h prior to the actual infection [31]. 2 h prior to infection, 50 nl of compound in DMSO (0.1% final DMSO concentration per well) were transferred with a PinTool (GNF Systems) into the assay plates (10 µM final concentration). *P. yoelii* sporozoites were freshly dissected from infected *A. stephensi* mosquito salivary glands and filtered twice with a 40-µm strainer. The HepG2 cells were then infected with 8×10^3^ sporozoites per well. After infection and 1-h incubation at 37°C, the cultures were washed, new media and compound added, and further incubated with 5-fold increased concentration of penicillin/streptomycin for 48 h at 37°C before exoerythrocytic forms (EEFs) quantification of infected cells by immunofluorescence.

#### EEF immunofluorescence quantification

After washing with 1×PBS and fixation of the cells with 4% paraformaldehyde solution (EMS), membranes were permeabilised with 0.5% Triton-X-100 (Thermo Fisher Scientific) and EEFs were stained using a mouse polyclonal serum raised against the *Plasmodium* heat shock protein 70 (HSP70), an Alexa goat antimouse IgG, Fca-specific DyLight 649 secondary antibody (Invitrogen), and the Hoechst 33342 nucleic acid dye (Invitrogen). Stained EEFs were then quantified using the Opera Confocal High Content Screening system (PerkinElmer). Images were collected using a 20× magnification at a binning of 2 using a 365 Xeon arc lamp illumination to detect the nuclei and 635-nm laser line to excite DyLight649.

High-content imaging of infected HepG2-CD81 cells was performed as described in Meister et al. [31]. Wells were analysed using a custom Acapella (PerkinElmer) script parameterized for this assay. In brief, images from fields inside the well were first discarded as out of focus when the intensity in the nuclear area was too low. Then hepatic cells were counted by detecting the nuclei labelled with Hoechst 33342 and parasites were segmented using the HSP70 immuno-labelling. Morphology-based (e.g., area, roundness) and intensity-based features were calculated for each object detected including the hepatocyte nuclei and the parasites. Parasitemia was set as the ratio between parasite number (Alexa fluor positive) and the hepatocyte nuclei count, determined at the same time.

## Results

### Schizonticidal Activities of Compounds against Strains of *P. falciparum* with Known Drug Resistance Markers

**Figure 1:**
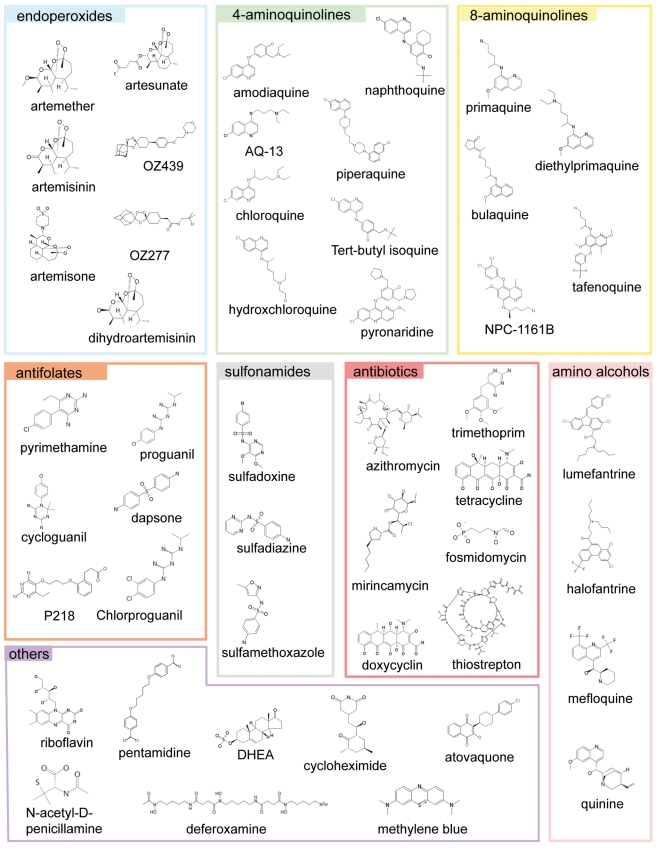
The main classes of antimalarials. The chemical structures of all the main classes of antimalarials and other therapeutic and control molecules are assembled according to either the chemical classes they belong to (endoperoxides, 4- and 8- AQs, amino-alcohols) or their function (antifolate, antibiotics), or both (e.g., sulfonamides, a chemical class of antibiotic used in combined antimalarial therapies). The colour code associated with each class is consistent in all the figures in this report.

**Figure 2:**
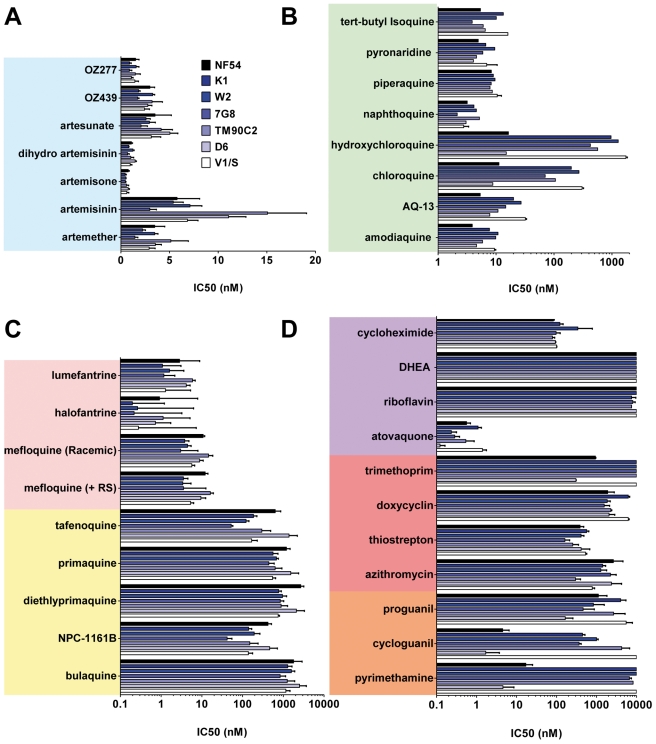
The potencies of selected antimalarials against asexual blood stages. Main classes of antimalarials were tested against the blood stage of seven *Plasmodium falciparum* strains in the [^3^H]hypoxanthine incorporation assay as described in Vennerstrom et al. [25]. The strains tested here (obtained from MR4) were two drug-sensitive strains NF54 and D6 and five drug-resistant strains: K1, resistant to chloroquine (CQ) and pyrimethamine (PYR), origin Thailand, carries mutations in genes pfmdr1, pfcrt, pfdhfr, pfdhps; W2, resistant to CQ, PYR, quinine, cycloguanil, and sulfadoxine, origin Vietnam; 7G8, resistant to CQ and PYR, origin Brazil; TM90C2A, resistant to CQ, PYR, and MFQ, origin Thailand; and V1/S, resistant to CQ, PYR and cycloguanil, origin Vietnam. Results are expressed as the concentration resulting in 50% growth inhibition (IC_50_).Values are means of ≥3 independent experiments. (A) Endoperoxides. (B) 4-AQs. (C) 8-AQs and amino alcohols. (D) Antifolates, antibiotics, and others.

A collection containing all antimalarials approved for use in humans and those in clinical development, anti-infectives, and other controls (Figure 1) was profiled simultaneously on asexual blood stage parasites in a standardized growth inhibition assay (GIA) [25] using seven strains of *P. falciparum* exhibiting diversity in the molecular causes of resistance and geographical origins (Table S1). The half-maximal inhibitory concentrations (IC_50_) were determined for each molecule (Table S2). Known mutations in *pfcrt*, *pfmdr1*, *pfdhfr*, and *pfdhps* correlated with a loss of potency of at least 10-fold for the relevant drugs. As expected the 4-aminoquinolines (4-AQs) chloroquine and hydroxychloroquine showed a 6–100-fold reduction in potency against all drug-resistant strains containing the mutated chloroquine transporter (PfCRT) (Figure 2B). Amodiaquine, tert-butyl-isoquine pyronaridine, piperaquine, and naphthoquine were potent against all parasite lines (IC_50_ = 2–10 nM), as were the endoperoxides including the natural (artemisinin), the semi-synthetic (artesunate), or the fully synthetic peroxides (ozonides), all with IC_50_ values of 1–15 nM (Figure 2A).

Compared to the 4-AQs, the 8-AQs (8-AQs) primaquine, diethylprimaquine, and bulaquine were less potent (IC_50_ = 0.5–2.5 µM) (Figure 2C) against both drug-resistant and -sensitive strains. Tafenoquine and NPC-1161B exhibited IC_50_ values in the 500-nM range against NF54 and in the 50-nM range against 7G8. IC_50_ values of both racemic mefloquine and the +RS isomer were below 10 nM. Halofantrine and lumefantrine displayed potencies below 4 nM against the sensitive strains NF54 and D6 and in the case of halofantrine, 0.3 nM against the multi–drug-resistant strains K1, W2, and 7G8. Resistance to pyrimethamine was verified in all drug-resistant strains (Figure 2D). Atovaquone a drug active in the subnanomolar range against *P. falciparum* blood stages showed at most a 10-fold difference in potency between the strains D6 and V1/S. In contrast, a ≥100-fold loss of potency was observed for cycloguanil between sensitive and resistant strains. The antibiotics azithromycin and trimethoprim, protein synthesis inhibitors, and other molecules such as dehydroepiandrosterone (DHEA), riboflavin, doxycyclin, and the prodrug proguanil showed IC_50_ values in the 1–10-µM range with no major differences between strains.

### The Identification of Drugs That Additionally Block Transmission from Human Host to Mosquito Vector

**Figure 3:**
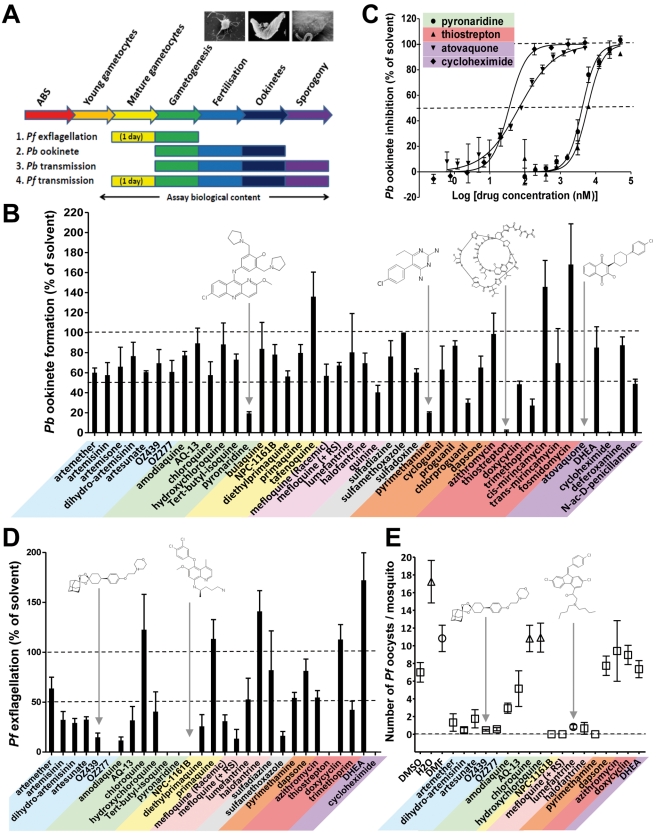
The transmission-blocking potential of selected antimalarials in three bioassays that cover different phases of *Plasmodium* vector stage development. (A) Assays examined exflagellation (*P. falciparum*), ookinete formation (*P. berghei*), or oocyst formation (*P. falciparum*). All antimalarials were screened at 10 µM. (B) The biological content of the in vitro *P. berghei* ookinete assay spans gamete formation, fertilization, zygote development, and ookinete formation. Ookinete formation was insensitive to most of the antimalarials tested. Atovaquone, cycloheximide, pyronaridine, pyrimethamine, and thiostrepton all strongly inhibited ookinete formation (*p*<0.03 by Student's *t*-test), while tafenoquine, cis-mirincamycin, and fosmidomycin gave an enhancement of ookinete formation that was not statistically significant. (C) The medium throughput fluorescent ookinete assay determined IC_50_ values for pyronaridine, thiostrepton, cycloheximide, and atovaquone of 6 µM, 8 µM, 25 nM, and 65 nM, respectively. (D) The in vitro *P. falciparum* exflagellation assay exposes mature gametocytes to antimalarials for 24 h before triggering exflagellation. 16 out of 29 antimalarials tested, including all but one of the endoperoxides and 4-AQs, showed a statistically significant >50% inhibition of exflagellation (*p*<0.05). Pyronaridine, tert-butyl isoquine, NPC-1161B, OZ277, and cycloheximide all inhibited exflagellation totally at 10 µM. All experiments in triplicate. (E) The in vivo *P. falciparum* oocyst assay differs from the *P. berghei* oocyst assay in that mature gametocytes were exposed to the antimalarials in culture for 24 h before feeding to mosquitoes. The endoperoxides all strongly reduced transmission. NPC1161-B, lumefantrine, halofantrine, and mefloquine +RS isomer were also active. (*n* = 4–61 observations; average ± standard error of the mean [SEM]).

Eradicating malaria will require medicines that prevent transmission of the parasite between humans and mosquitoes. Potentially the severe population bottleneck experienced as the parasite progresses from the mature gametocyte in the human host through gametogenesis and fertilization in the mosquito blood meal to the oocyst in the mosquito haemocoele offers the most vulnerable target for intervention. We developed assays for each of these events (Figure 3A). To integrate both the early sexual stages (gametocyte maturation and gametogenesis) and the late vector stage (sporogony) into the drug-testing cascade, we measured the exflagellation of male gametes in vitro (*P. falciparum*), ookinete formation in vitro (*P.berghei*), and the production of oocysts in *A. stephensi* (*P. berghei* and *P. falciparum*). Of these assays, we found analysis of *P. berghei* ookinete production in vitro was the most robust approach to identify molecules potentially targeting the early development of *Plasmodium* parasites in the mosquito. Forty-six molecules were tested at a concentration of 10 µM (Figure 3B). The most potent molecules were cycloheximide (blood stage IC_50_ of 25 nM) and atovaquone (IC_50_ = 65 nM). Thiostrepton (IC_50_ = 8 µM) and pyronaridine (IC_50_ = 6 µM) were less potent (Figure 3C). The latter two molecules and pyrimethamine also inhibited *P. falciparum* exflagellation by more than 80%, as did sulfamethoxazole and mefloquine (+RS). While displaying insignificant activity in the *P. berghei* ookinete formation assay (Figure 3C), all endoperoxides, with the sole exception of artemether, inhibited *P. falciparum* exflagellation by >65% (Figure 3D). Similarly all 4-AQs inhibited this event by >60%, with the exception of hydroxychloroquine and chloroquine, which enhanced exflagellation by at least 20% [16].

While recognising that the drugs being evaluated are not subject to metabolic degradation/activation by the mammalian hosts, among the 8-AQs, NPC-1161B and diethylprimaquine inhibited exflagellation by more than 70%. In marked contrast primaquine and the amino alcohol halofantrine unexpectedly stimulated exflagellation by 15% and 45%, respectively. All main chemical classes were then evaluated specifically against the vector stages by analysing the inhibition of *P. berghei* oocyst formation in vivo. Only NPC-1161B and lumefantrine exhibited >90% inhibition when tested at 10 µM (Figure S1). The transmission-blocking potential of molecules that were active against exflagellation and/or *P. berghei* sporogony was then assessed against the production of *P. falciparum* oocysts, which is the most difficult and lowest throughput, yet highest content analysis by encompassing all vector stages from gametocyte uptake to sporogony (see Figure 3A). In this assay, most endoperoxides inhibited oocyst production by >75%; NPC-1161B and mefloquine (+RS) totally blocked transmission at this stage (Figure 3E). Strikingly, the Coartem component, lumefantrine, and halofantrine impaired sporogony in both *P. berghei* and *P. falciparum*, while both mediated only moderate or no inhibition of exflagellation. This finding suggests that these molecules might act specifically on oocysts and not on gametogenesis, a behaviour that could be relevant to transmission reduction in the field given the long half-life of these molecules. As exflagellation is a component process of development within the gut of the mosquito in the *P. falciparum* oocyst assay (see Figure S2), these results, not unexpectedly, show at least partial concordance with the *P. falciparum* exflagellation assay.

### The Identification of Drugs That Suppress Transmission from the Mosquito to the Human Host

**Figure 4:**
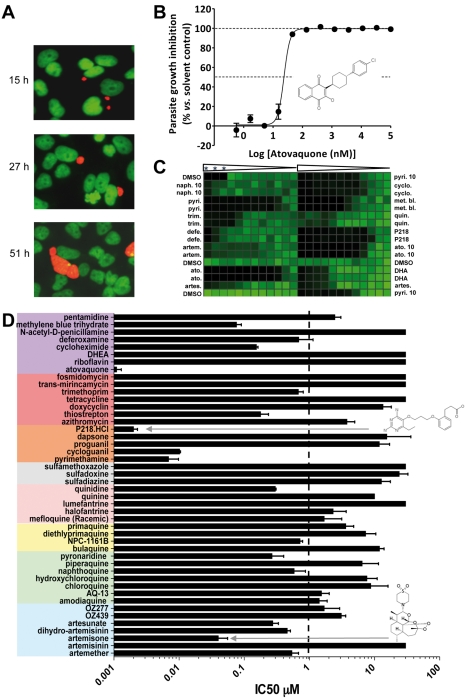
Validation and results of anti-infectives tested against *P. yoelii* liver stage parasites. (A) Opera-generated images from a time point experiment using a 20× objective lens. *P. yoelii* parasites (red) were visualized using a mouse polyclonal HSP70 antibody, and the HepG2 cells (green) were stained with Hoechst 33342 nucleic acid dye. Median parasite areas for the 15-h, 27-h, and 51-h time points were calculated as 16, 52, and 158 µm^2^, respectively, using a custom Acapella script. (B) A dose response plot with atovaquone from data generated from the Opera imaging system. The median areas of compound-treated parasites were compared to untreated DMSO controls to determine the degree of inhibition. The IC_50_ value of atovaquone is 22 nM. (C) From the Acumen ^e^X3 imaging system, a 384-well heat map plate image of *P.yoelii* fluorescence intensities from a dose response experiment with the most active anti-infectives. The three wells at the top left corner were not infected with sporozoites and served as a negative control. Wells were treated with a 1∶3 dilution of compounds starting at 30 µM (0.1% DMSO). In the experiments where the highest compound concentration tested was 10 µM, the entry is labelled 10. Compounds used are: naphthoquine (naph.), pyrimethamine (pyri.), trimethoprim (trim.), deferoxamine (defe.), artemisone (artem.), atovaquone (ato.), artesunate (artes.), cycloguanil (cyclo.), methylene blue (met. bl.), quinidine (quin.), dihydroartemisinin (DHA). Triangles represent the dilution steps of the drugs as described above. Stars designate the three wells with no sporozoite. (D) IC_50_ results from a compound dose response experiment performed in a 384-well plate format. Results were generated from the Opera using median parasite area to determine level of inhibition.

When an infected mosquito bites a host, ∼100 sporozoites may be injected into the dermis from where they rapidly invade liver cells [32],[33]. This infective step represents the second bottleneck during transmission and therefore another potentially vulnerable point for intervention. In the absence of a practical liver stage assay measuring the formation of *P. falciparum*/*P. vivax* liver schizonts, an equivalent assay was developed in *P. yoelii* and used to assess the activity of the collection of molecules against this specific stage. Specifically, *P. yoelii* sporozoites were dissected from the salivary glands of infected mosquitoes and were allowed to invade human hepatocarcinoma cells expressing the CD81 protein. The development of the liver schizonts was monitored by immunofluorescence staining using an HSP70 antibody specific to the parasite (Figure 4A). As only 1% of the hepatocytes are infected in these circumstances, high content imaging was used to quantify growth inhibition of parasite schizonts (Figure 4B, 4D). Quantification of the total immunofluorescence per well is shown in Figure 4C. Dose response analysis using serially diluted compounds showed that cycloguanil, pyrimethamine, P218, and atovaquone all displayed IC_50_ values below 10 nM (Figure 4B, 4D). Methylene blue and artemisone demonstrated IC_50_ values of <100 nM. All endoperoxides tested in this assay, except artemisinin, exhibited IC_50_ values <3 µM, as did amodiaquine, AQ-13, pyronaridine, and naphthoquine. Of the 8-AQs, NPC-1161B was the only drug active in the submicromolar range against *P. yoelii* liver stages. Deferoxamine, thiostrepton, trimethoprim, and quinidine exhibited submicromolar potencies. Cycloheximide and thiostrepton, although showing IC_50_ values below 200 nM (Table 1), retarded the growth of HepG2-CD81 cells.

**Table 1:** The ability of key molecules to provoke growth retardation of HepG2 cells.

Molecules	IC_50_ Parasite (µM)	IC_50_ HepG2 (µM)	CI
Halofantrine	2.32±1.35	<5.66±8.00	<2.44
Mefloquine	1.69±1.45	1.73±0.60	1.02
NPC-1161B	0.72±0.07	0.84±0.46	1.17
OZ277	1.69±1.22	7.40±5.13	4.38
Pyronaridine	0.27±0.13	<0.17±0.25	<0.63
Thiostrepton	0.18±0.05	0.18±0.02	1.0
Cycloheximide	0.15±0.01	0.92±0.29	6.13
Proguanil	11.80±5.19	15.44±3.09	1.31
Pentamidine	2.46±0.57	3.75±1.10	1.52

Data were compared to the antimalarial potency of the same molecules against the development of *P. yoelii* liver schizonts following infection of HepG2 cells.

CI, cytotoxicity index.

## Discussion

### Modes of Action of Major Classes of Antimalarials beyond the Asexual Blood Stage

The IC_50_ values of the known schizonticides against the asexual blood stages determined in this study correlate well with those reported in recent work examining 185 culture-adapted parasite strain lines treated with seven antimalarials [34].

Many schizonticidal drugs are hypothesised to interfere with haemoglobin metabolism. Our study shows that some of these drugs have activities against parasite stages that lack haemoglobin metabolism, e.g., the liver schizont, mature gametocyte, and sporogonic stages. This finding raises interesting questions about the mode(s) of action of these compounds beyond the asexual blood stage and our understanding of parasite metabolism. For example, natural, semi-synthetic and synthetic endoperoxides (artemisinin, DHA, artesunate, OZ277, and OZ439) are not only fast-acting molecules [35], but are among the most potent antimalarials currently used against the asexual blood stages [36]. They are thought to act by alkylating haem and other vital biomolecules [37],[38] (e.g., *Pf* TCTP) [39], and degrading phospholipids in parasite membranes [40]. The latter mechanism would be expected to have a major impact on all vegetative/replicating stages of *Plasmodium's* life cycle, e.g., asexual blood stage, liver schizont, oocyst, and microgametogenesis; and this is consistent with our results showing endoperoxide activity directly or indirectly against *P. falciparum* exflagellation, oocyst production, and *P. yoelii* liver schizont development. The lesser impact of this chemical class on ookinete and oocyst development in *P. berghei* might suggest species to species differences. It is also worth noting that because drugs are applied in human blood containing mature gametocytes prior to triggering exflagellation, inhibition of exflagellation by these molecules could be the direct consequence of a gametocytocidal property rather than solely a specific effect on gamete formation. Our data confirmed that 4-AQs are highly active against asexual blood-stage parasites in vitro, while the 8-AQs are not, indicating that subtle changes in the AQ core structure can result in major differences in the mode of action of some key antimalarials, although our data cannot address the possibility that the 8-AQ metabolites could be more active. Interestingly, molecules such as amodiaquine, pyronaridine, and tert-butyl-isoquine that target inter alia haem degradation also inhibited *P. falciparum* exflagellation. We are unaware of any prior data suggesting that haem degradation is essential to male gamete formation. This finding raises fascinating questions as to whether their modes of action in the mosquito blood meal are targeting the same, or different, molecular mechanisms. One working hypothesis could be that the major targets mediating the effect of these molecules in the sexual stages might also contribute to the effect seen in the asexual stages.

The 8-AQs are known to be active on the relapsing “hypnozoite” liver form of *P. vivax* following metabolic activation of the parent compound by liver enzymes [41]. In our assay where we do not anticipate any metabolism of any drugs, we note that the 8-AQ NPC-1161B was additionally shown to inhibit exflagellation in vitro and oocyst production in the mosquito vector. This result suggests either that NPC-1161B does not require metabolic activation or the drug exhibits poly-pharmacology by acting through another metabolite-independent mechanism. The antifolates P218 and pyrimethamine were found to be potent against rapidly replicating blood and liver stages and much less so against early vector stages, observations consistent with the essential role of the folate pathway in DNA synthesis [42].

When mature gametocytes are ingested, exflagellation is activated by a reduction in temperature and the presence of xanthurenic acid in the gut of the mosquito [43]. Inhibition of exflagellation by thiostrepton and cycloheximide is consistent with the observation that protein synthesis is a key component of these dramatic morphological changes [27], and all vegetative stages in the life cycle. Similarly our data confirm previous studies [44]–[46] showing the electron transport chain can be efficiently targeted by atovaquone in both the vector and the liver. For such stage-transcendent pathways, it could be hypothesised that the much lower parasite burden observed in the vector and the liver would increase the probability that treatments would eliminate infections when compared to targeting the abundant blood stage parasites. An important consequence would be that drugs specifically targeting the small “bottleneck” populations might delay significantly the selection of drug-resistant parasites.

### “Management” of Drug Delivery to the Mosquito Blood Meal

While both our studies and the comprehensive treatise of Peters [47] show unequivocally that for many compounds effective delivery into the blood meal of the mosquito can be achieved, management of such delivery in the field is not trivial. In this context we note that the C_max_ following administration of therapeutic doses for many antimalarials overlap the predicted potencies of the same antimalarials determined here against early vector stages (0.5–10 µM). For instance, in reported clinical trials, C_max_ was shown to vary from 0.1 µM to 13.9 µM for artemether and lumefantrine administered at therapeutic doses, respectively. In the case of new molecules such as OZ439, C_max_ values obtained in blood at relevant doses ranged between 1 and 2.5 µM (Table S3) [48]–[55].

### Combination Therapies

To support the eradication agenda, new combination therapies will have to address three major issues: transmission of the pathogen, radical cure of *P. vivax* malaria, and the emergence of drug resistance. Ideally, new drug combinations should contain both schizonticidal and transmission-blocking components. Preferably fast-acting and long-lasting schizonticides would be combined with another agent that would target the parasite either at the sexual/vector stages, liver stage, or both. In the case of the liver stage, a co- or post-treatment prophylaxis would be provided as an end game scenario. From a pharmacokinetic perspective, when considering *P. falciparum*, some might speculate that this additional transmission-blocking component should be stable enough to exert its inhibitory capacity over several days against vector stages and/or liver schizont development to protect against a re-infection. However, considering that the schizonticidal component would kill both asexual blood stages and young gametocytes (<6-d old), a second coadministered drug efficiently eliminating mature (>6-d) gametocytes could potentially clear the host of all parasites; i.e., a long half-life would not be required [56]. A sustained stability would however be required for drugs targeting the parasite exclusively in the mosquito. Our study suggests that molecules such as atovaquone that inhibit the electron transport chain in the parasite mitochondria could be suitable candidates, but ideally should lack delayed onset of action and be difficult to raise resistance against [57]. To avoid triple therapies and reduce the risk of drug resistance, dual-activity molecules like amodiaquine, which inhibits haemoglobin digestion in the asexual blood stages and potentially inhibits gametocyte maturation/gamete exflagellation by a different mechanism, could be used in combination with a second antimalarial. Such polyvalent multistage activity has significant benefit to overall drug impact. Our study highlights that molecules such as amodiaquine, naphthoquine, tert-butyl-isoquine, and piperaquine do not lose potency when tested against chloroquine resistant strains. Therefore, to mitigate or defer the risk of drug resistance these molecules might be proposed as potential candidates for partnering new antimalarials such as OZ439. An important consideration would be to favour molecules that have never been used as monotherapy to avoid facing parasites that have previously acquired drug resistance. NPC-1161B inhibited both exflagellation and oocyst production; new molecules with similar properties but devoid of haemolytic liability of the 8-AQs in glucose-6-phosphate dehydrogenase (G6PD)–deficient patients could be interesting candidates as specific transmission-blocking agents.

While being strong inhibitors of blood-stage parasites, some molecules such as chloroquine reportedly enhance gametocytogenesis—a property that might have facilitated the spread of drug-resistant parasites [15],[58]. The panel of assays that we applied in this study has confirmed such “collateral” activity, in this case one that could prejudice the rational implementation in any elimination/eradication strategy.

### Other Human Malarias

Although many have argued for the use of drug screens using nonhuman malarias [47], we recognize that to some, including assays with rodent malaria species might be considered suboptimal. Recent observations identifying interspecific variations include cysteine proteases in rodent plasmodia that show subtle active site differences to those in *P. falciparum*, leading to questions about the use of these models [59]. A critical role of amino acid 23 mediates activity and specificity of vinckepain-2, a papain-family cysteine protease of rodent malaria parasites [60]. *P. yoelii* 17X strain is intrinsically partially resistant to chloroquine and is therefore a poor model for studying acquisition of *P. falciparum* chloroquine resistance. Nevertheless we must recall that there are five species of *Plasmodium* that infect man and their biologies are patently different, therefore detecting drugs that may be active against multiple species in initial screens may offer long-term potential. Our assays provide, to our knowledge, the most comprehensive global overview of antimalarial drug action to date within the constraints imposed by the current state of culture methodologies for all life stages of all mammalian malaria parasites. Ideally, antimalarials developed against *P. falciparum* would have an even broader clinical usefulness if proven to be as effective against *P. vivax* [61]. The potencies of some antimalarials against the asexual blood stage of cultivated *P. falciparum* and *P. vivax* field isolates show a very good correlation (Table S4) [62]–[67]. This observation suggests that most of the pathways inhibited by antimalarials in *P. falciparum* are conserved and may offer valid targets in *P. vivax*. Moreover, the endoperoxide OZ439, which is currently evaluated in phase IIa clinical trials, has recently demonstrated equivalent efficacy in the treatment of *P. falciparum* and *P. vivax* patients (personal communication, MMV).

### Drug Gaps and Future Steps

Our work has revealed previously unforeseen opportunities in the current discovery and development pipeline for new antimalarials. We demonstrated that drugs in the current portfolio, like pyronaridine and atovaquone, can also target liver and sexual stages in addition to asexual blood stages. Safe and stable drugs with similar multistage potential are now required. Developing drugs with long half-lives like mefloquine and chloroquine is essential to ensure that in patient blood the exposure of these drugs will remain above the minimum inhibitory concentration for several erythrocytic cycles and should ideally cover the period of gametocyte maturation. Additionally, new chemical scaffolds (e.g., nonendoperoxide) with fast killing potential are needed for a “one dose cure.”

The search for new drugs would be enhanced by the continued development of *P. falciparum* and *P. vivax* culture systems for every parasite life stage. It is critical to the malaria eradication agenda that these assays are able to identify drugs, such as the 8-AQs, with the capacity to safely eliminate *P. vivax* hypnozoites from the liver: this objective will also require the implementation and validation of in vitro and in vivo G6PD deficiency-dependent hemolysis assays. In parallel with efforts to discover innovative drugs for radical cure (elimination of *P. vivax* hypnozoites from the liver), new molecules blocking the onward development of mature (stage V) gametocytes are the other major priority in antimalarial discovery. There is therefore an urgent need to develop and validate high throughput screening assays allowing new libraries to be tested against *P. falciparum* and *P. vivax* gametocytes/transmission. These assays could then prioritise compounds for examination in preclinical studies in small mammals and then in standard membrane feeding assays (SMFA) using patient blood to find drugs blocking transmission in the field/clinical situation. Fields studies will remain essential to carefully examine any correlation between activities of new molecules against stabilized laboratory parasite strains and against field isolates.

### Conclusions

**Figure 5:**
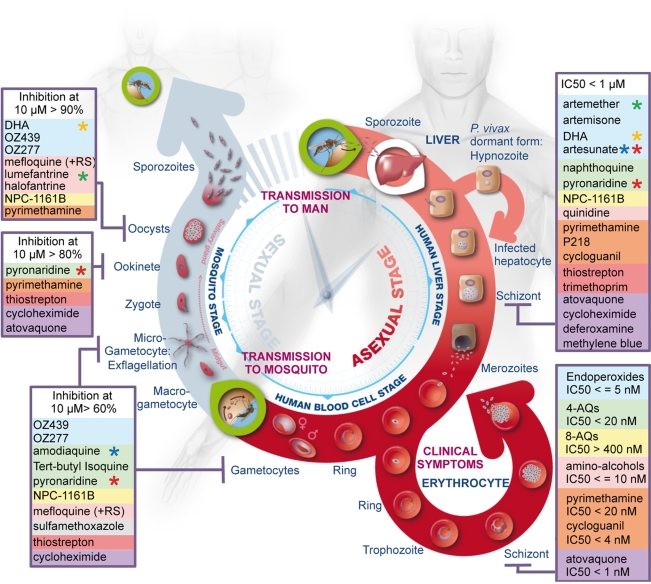
Summary of the activity of the most widely used antimalarials throughout the life cycle of *Plasmodium*. The three main phases, i.e., liver stage, blood stage, and vector stage, of the life cycle of *Plasmodium* are shown. The two key entry points leading to transmission of the parasites from vector to host and from host to vector are indicated (green circles). Parasite forms specific to each stage are highlighted and drugs identified as inhibitors of development of these forms are listed in boxes and coloured as described in Figure 1. Stars highlight components of the main artemisinin combination therapies: green, coartem; red, pyramax; orange, eurartesim; blue, ASAQ.

For the first time, the main chemical classes of current and future antimalarials have been profiled simultaneously in standardized conditions against three *Plasmodium* species with respect to every major cellular strategy of the malarial life cycle, e.g., vegetative replication, dispersal, and sex (Figure 5). The present study provides the antimalarial research community with a reference set of methods and data, which may serve as a benchmark for newly discovered molecules when profiled against the entire life cycle of *Plasmodium*. This information might guide decisions regarding which molecules could be optimally combined to provide the next generation of drugs that will succeed to artemisinin combination therapies (ACTs) [68] and support the eradication of malaria. This comprehensive approach to drug discovery has potential utility for targeting other pathogens with complex life cycles.

## Supporting Information

Figure S1 **The transmission-blocking potential of selected compounds against** ***P. berghei*** **in standard membrane feeding assays.** All antimalarials were screened at 10 µM in triplicate in independent experiments. The biological content of this assay spans gamete formation through to occyst development all within the gut of the mosquito. NPC1161-B, lumefantrine, pyrimethamine, and cycloheximide showed most notable transmission-blocking activity.
(TIF)

Figure S2 **Comparative summary of the transmission-blocking potential of selected compounds across vector-stage assays.** All antimalarials were screened at 10 µM. By comparing the activities of compounds in assays covering different biological ranges of transmission-stage biology, it is possible to infer the stages at which antimalarial drugs are exerting their effects. Lumefantrine was found to have little activity against exflagellation and ookinete development but showed activity in oocyst assays of *P. berghei* and *P. falciparum*. Endoperoxides showed activity as early as exflagellation but not during ookinete development, indicative of action against the mature gametocyte/exflagellation. NPC1161-B showed potency in all assays except the ookinete assay, suggesting that it may have dual actions both in early vector-stage development and later on.
(TIF)

Table S1 **The origin and reported drug resistance of** ***P. falciparum*** **strains used in this study.** CQ, chloroquine; PYR, pyrimethamine; CYC, cycloganil; QUI, quinine; SUL, sulfadoxine; MFQ, mefloquine; ATO, atovaquone.
(PPT)

Table S2 **The numerical potencies of selected antimalarials against asexual blood stages.** Data corresponding to Figure 2, showing the numerical IC_50_ values of selected antimalarial compounds against seven *P. falciparum* strains in the [^3^H]hypoxanthine incorporation assay.
(TIF)

Table S3 **A comparison of antimalarial exposure in human blood against predicted early vector-stage potency.** The IC_50_ values of selected antimalarials in the *P. falciparum* exflagellation assay was estimated using additional data generated by screening at 1 µM (*) and compared to drug Cmax values obtained from the literature. The in vitro assay contains both blood and serum. Most of the cells in culture (∼97%) are uninfected RBCs. The medium that the assay is set up in is derived from RPMI and contains 10% human serum.
(TIF)

Table S4 **A comparison of the reported asexual blood stage potencies of selected antimalarials against** ***P. falciparum*** **(field and laboratory isolates) and** ***P. vivax*** **(field isolates).**
(TIF)

## Abbreviations

AQ: aminoquinoline
RBC: red blood cell
EEF: exo-erythrocytic form
