# Supplementary figures and images for: The Activities of Current Antimalarial Drugs on the Life Cycle Stages of Plasmodium: A Comparative Study with Human and Rodent Parasites
Source: PLoS Med. 2012 Feb 21;9(2):e1001169. doi: 10.1371/journal.pmed.1001169 (PMC3283556; doi:10.1371/journal.pmed.1001169)

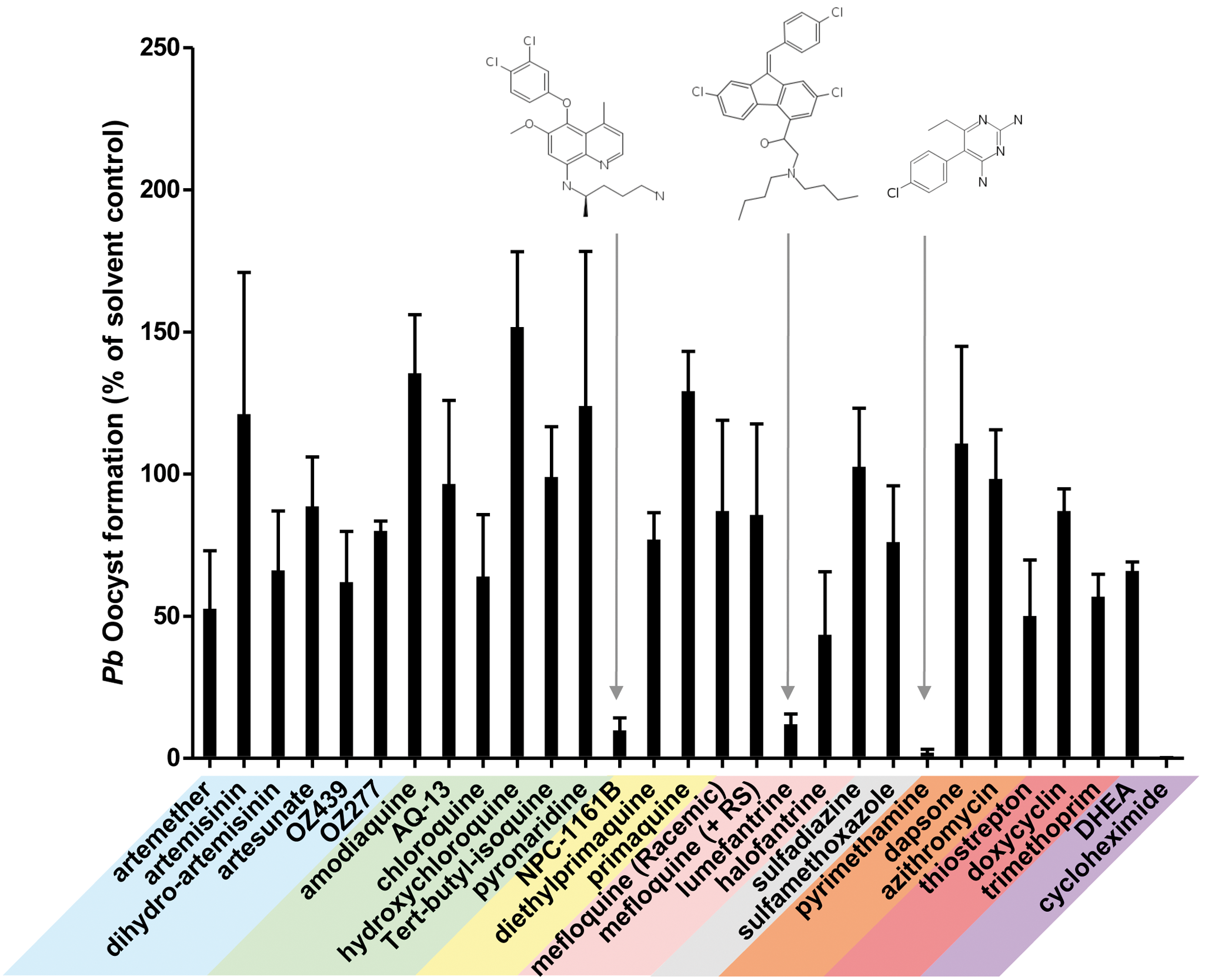

Supplement: Figure S1 — The transmission-blocking potential of selected compounds against P. berghei in standard membrane feeding assays. All antimalarials were screened at 10 µM in triplicate in independent experiments. The biological content of this assay spans gamete formation through to occyst development all within the gut of the mosquito. NPC1161-B, lumefantrine, pyrimethamine, and cycloheximide showed most notable transmission-blocking activity. (TIF) [file pmed.1001169.s001.tif]

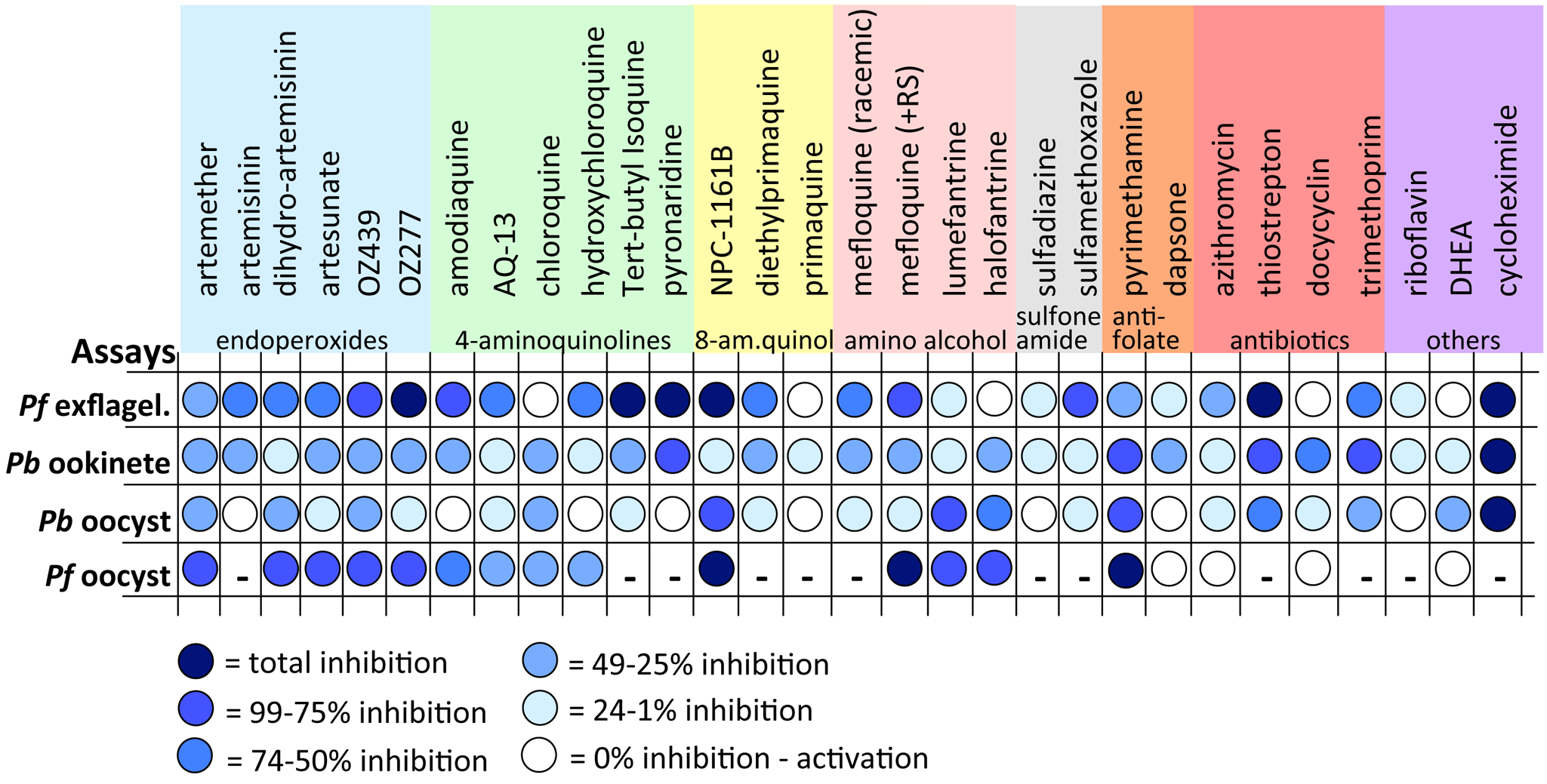

Supplement: Figure S2 — Comparative summary of the transmission-blocking potential of selected compounds across vector-stage assays. All antimalarials were screened at 10 µM. By comparing the activities of compounds in assays covering different biological ranges of transmission-stage biology, it is possible to infer the stages at which antimalarial drugs are exerting their effects. Lumefantrine was found to have little activity against exflagellation and ookinete development but showed activity in oocyst assays of P. berghei and P. falciparum. Endoperoxides showed activity as early as exflagellation but not during ookinete development, indicative of action against the mature gametocyte/exflagellation. NPC1161-B showed potency in all assays except the ookinete assay, suggesting that it may have dual actions both in early vector-stage development and later on. (TIF) [file pmed.1001169.s002.tif]

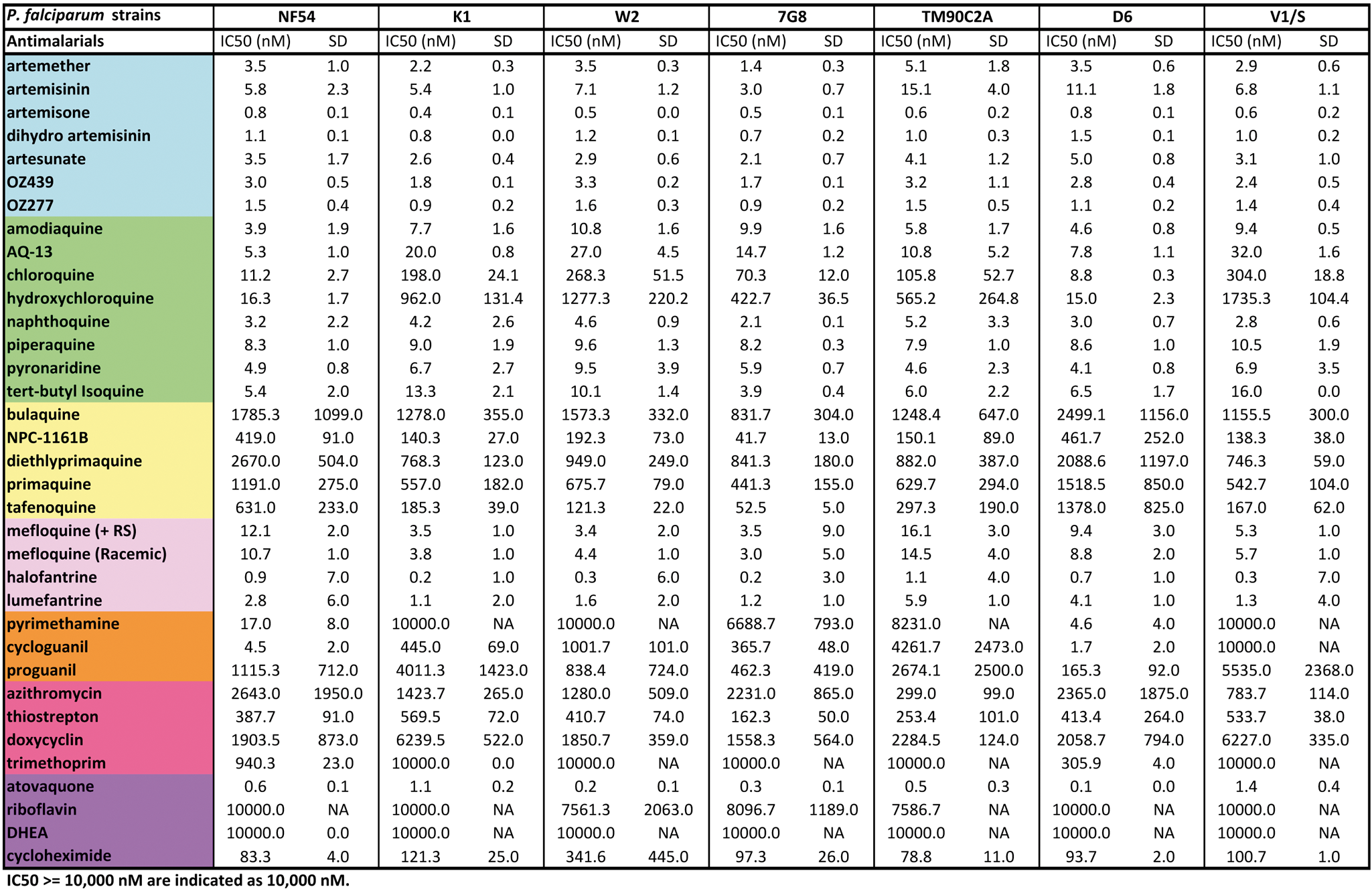

Supplement: Table S2 — The numerical potencies of selected antimalarials against asexual blood stages. Data corresponding to Figure 2, showing the numerical IC50 values of selected antimalarial compounds against seven P. falciparum strains in the [3H]hypoxanthine incorporation assay. (TIF) [file pmed.1001169.s004.tif]

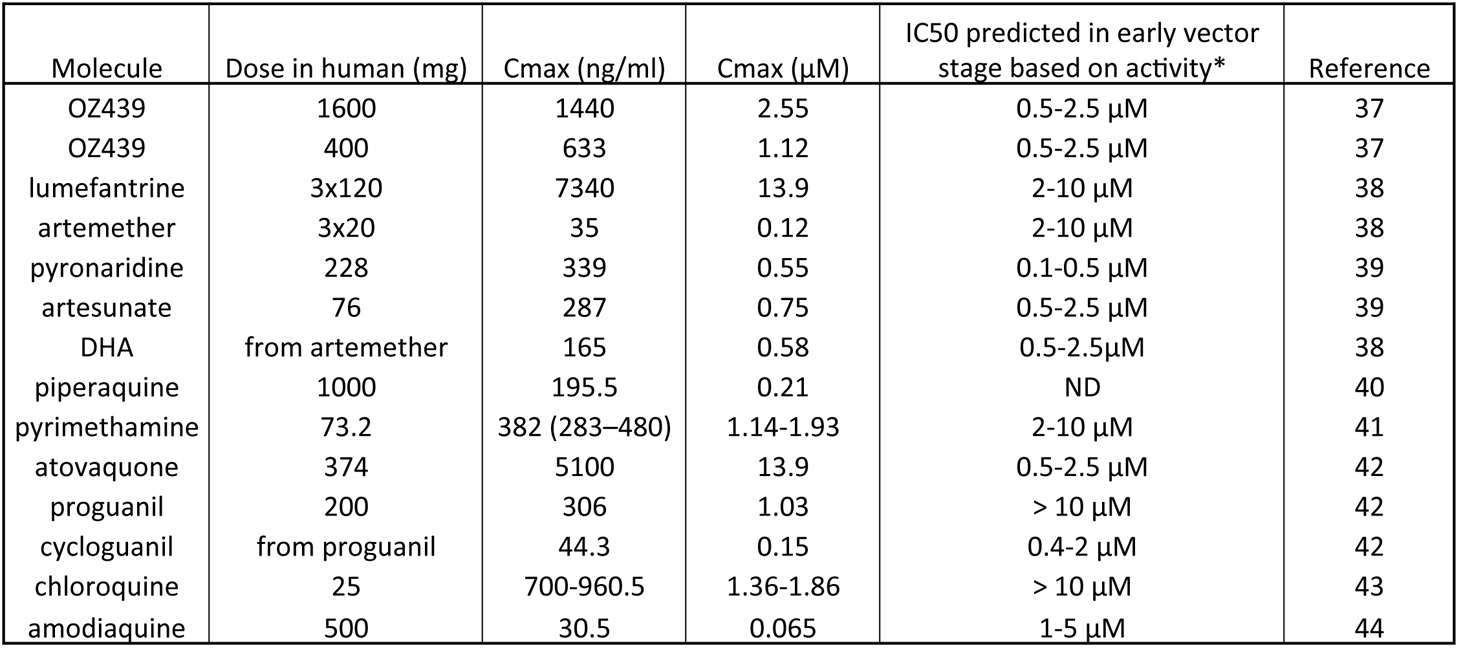

Supplement: Table S3 — A comparison of antimalarial exposure in human blood against predicted early vector-stage potency. The IC50 values of selected antimalarials in the P. falciparum exflagellation assay was estimated using additional data generated by screening at 1 µM (*) and compared to drug Cmax values obtained from the literature. The in vitro assay contains both blood and serum. Most of the cells in culture (∼97%) are uninfected RBCs. The medium that the assay is set up in is derived from RPMI and contains 10% human serum. (TIF) [file pmed.1001169.s005.tif]

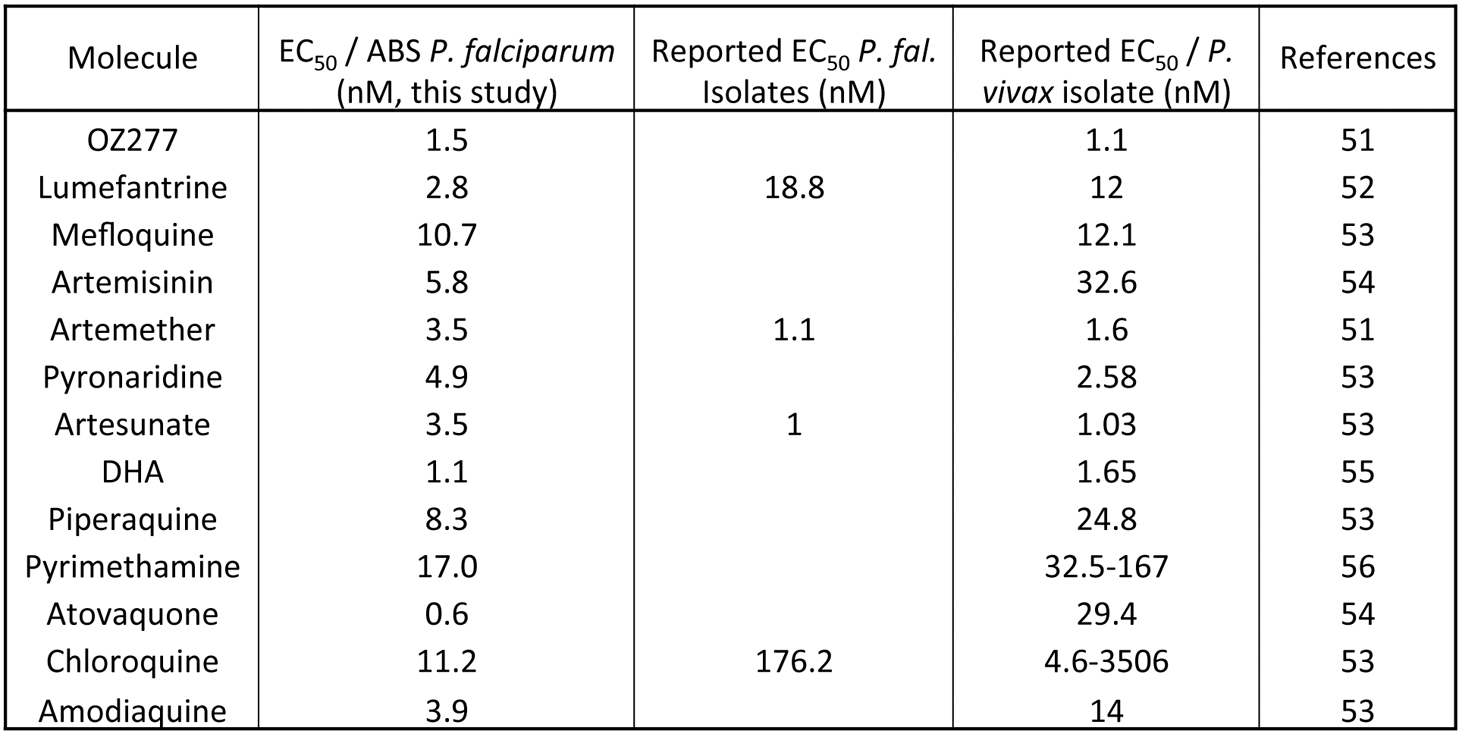

Supplement: Table S4 — A comparison of the reported asexual blood stage potencies of selected antimalarials against P. falciparum (field and laboratory isolates) and P. vivax (field isolates). (TIF) [file pmed.1001169.s006.tif]
