# Supplementary material for: The Activities of Current Antimalarial Drugs on the Life Cycle Stages of Plasmodium: A Comparative Study with Human and Rodent Parasites
Source: PLoS Med. 2012 Feb 21;9(2):e1001169. doi: 10.1371/journal.pmed.1001169 (PMC3283556; doi:10.1371/journal.pmed.1001169)
Supplement: Table S1 — The origin and reported drug resistance of P. falciparum strains used in this study. CQ, chloroquine; PYR, pyrimethamine; CYC, cycloganil; QUI, quinine; SUL, sulfadoxine; MFQ, mefloquine; ATO, atovaquone. (PPT) [file pmed.1001169.s003.ppt]

## Slide 1
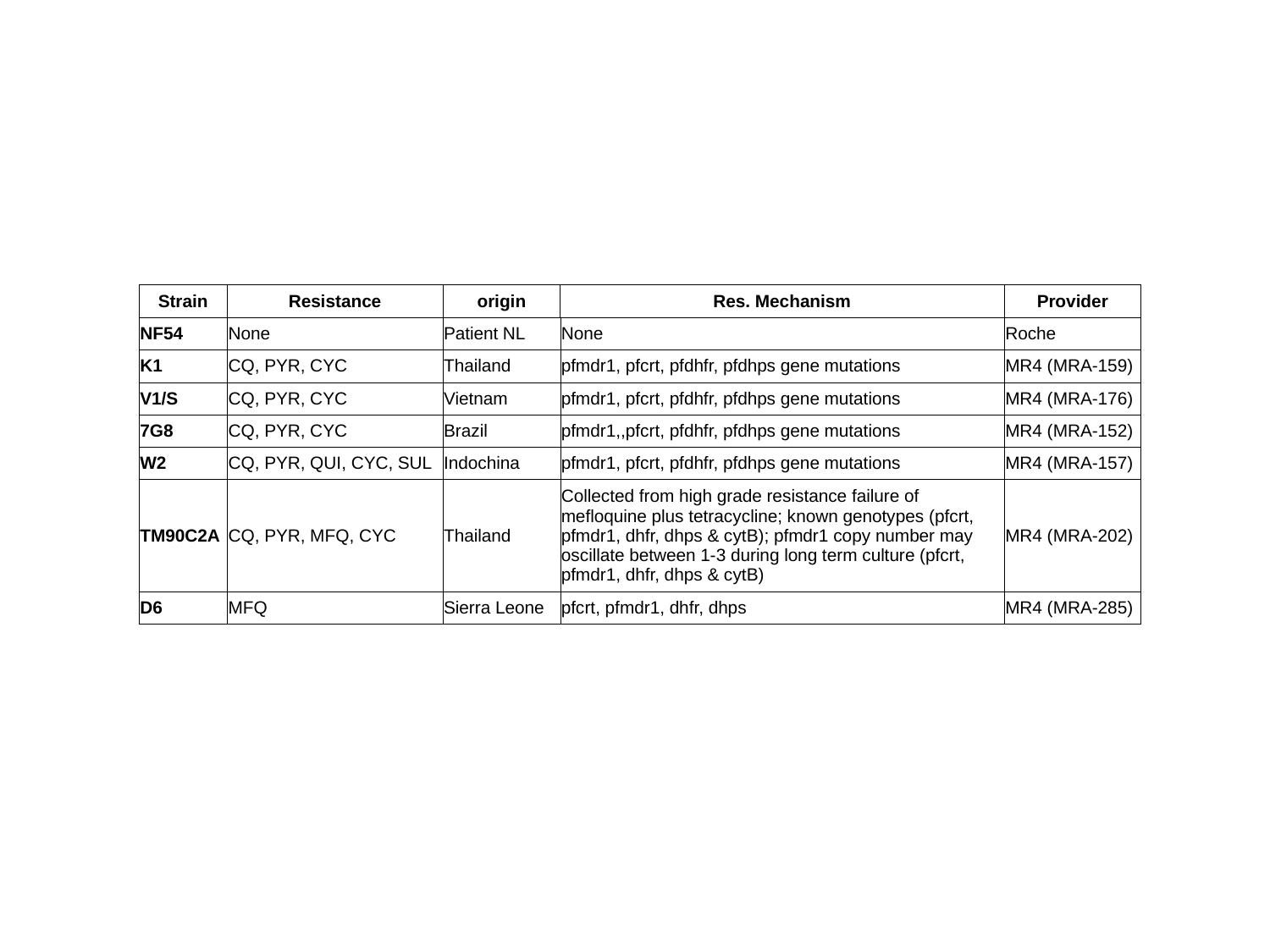

| | | | | |
| --- | --- | --- | --- | --- |
| | | | | |
| Strain | Resistance | origin | Res. Mechanism | Provider |
| NF54 | None | Patient NL | None | Roche |
| K1 | CQ, PYR, CYC | Thailand | pfmdr1, pfcrt, pfdhfr, pfdhps gene mutations | MR4 (MRA-159) |
| V1/S | CQ, PYR, CYC | Vietnam | pfmdr1, pfcrt, pfdhfr, pfdhps gene mutations | MR4 (MRA-176) |
| 7G8 | CQ, PYR, CYC | Brazil | pfmdr1,,pfcrt, pfdhfr, pfdhps gene mutations | MR4 (MRA-152) |
| W2 | CQ, PYR, QUI, CYC, SUL | Indochina | pfmdr1, pfcrt, pfdhfr, pfdhps gene mutations | MR4 (MRA-157) |
| TM90C2A | CQ, PYR, MFQ, CYC | Thailand | Collected from high grade resistance failure of mefloquine plus tetracycline; known genotypes (pfcrt, pfmdr1, dhfr, dhps & cytB); pfmdr1 copy number may oscillate between 1-3 during long term culture (pfcrt, pfmdr1, dhfr, dhps & cytB) | MR4 (MRA-202) |
| D6 | MFQ | Sierra Leone | pfcrt, pfmdr1, dhfr, dhps | MR4 (MRA-285) |
